# Frailty and quality of life: a cross-sectional study of Brazilian patients with pre-dialysis chronic kidney disease

**DOI:** 10.1186/1477-7525-12-27

**Published:** 2014-02-28

**Authors:** Henrique Novais Mansur, Fernando AB Colugnati, Fabiane Rossi dos Santos Grincenkov, Marcus Gomes Bastos

**Affiliations:** 1 Federal University of Pernambuco, Academic Centre of Vitória, Alto do Reservatório Street, no number, Bela Vista, Vitória do Santo Antão, Pernambuco, Brazil; 2 IMEPEN Foundation, Federal University of Juiz de Fora, José Lourenço Kelmer Street, 1300, São Pedro, Juiz de Fora, Minas Gerais, Brazil; 3 Wolfgang Amadeus Mozart Street, 131, São Pedro, Juiz de Fora, Minas Gerais, Brazil

**Keywords:** Frailty, Aging, Quality of life, Chronic kidney disease, Fragilidade, Idosos, Qualidade de vida, Doença Renal crônica

## Abstract

**Purpose:**

Chronic kidney disease (CKD) induces frailty and worsens quality of life (QOL), even in the early stages of the disease and in young patients. However, there is a lack of knowledge about the relationship between frailty and QOL in CKD patients. Thus, we investigated this relationship in a sample of CKD patients.

**Methods:**

A cross-observational study was conducted, in which 61 CKD patients receiving pre-dialysis treatment were assessed. All participants completed the Short Form-36 Health Survey (SF-36). We used valid and reliable methods to classify subjects as frail or non-frail according to Johansen’s et al. (2007) criteria. A one-way analysis of variance (ANOVA) and chi-square tests were used to compare the groups. In addition, Spearman’s correlation analysis was conducted to measure associations between identified variables and frailty. We also performed simple linear regression using the SF-36 physical and mental composite scores.

**Results:**

Almost half of the sample (42.6%) exhibited evidence of frailty. The groups differed significantly in terms of age, gender, and all SF-36 domains, excluding Social Functioning and Role Emotional. Frailty was significantly associated with all SF-36 domains, again excluding Social Functioning and Role Emotional. Regression analysis revealed no significant between-group differences in composite physical and mental health scores generated by the SF-36 (p > 0.05).

**Conclusion:**

Frail and non-frail CKD patients differed significantly in seven of the eight SF-36 domains. The frail group displayed diminished physical and mental functioning when their SF-36 scores were divided by their physical and mental composite scores. Frailty was correlated with QOL domains, with the exception of the social domain. There is a need for interventions targeting the characteristics of frailty, to provide better treatment and optimize overall QOL.

## Abstract (pt)

**Abstract:**

**Introdução**

É conhecido que a doença renal crônica implica em pior qualidade de vida e em fragilidade, mesmo em fases iniciais da doença e em sujeitos mais jovens. Existe uma lacuna no conhecimento relacionando fragilidade e qualidade de vida em pacientes com doença renal crônica (DRC), em especial nos pacientes em pré-diálise. Objetivo: Investigar a qualidade de vida, a fragilidade e as possíveis relações entre elas, em uma população com DRC.

**Métodos**

Estudo observacional e transversal com 61 pacientes com DRC entre os estágios 3 a 5 em fase pré-dialítica. Os participantes responderam à escala do questionário SF-36, que se caracteriza por uma versão reduzida do questionário da *Medical Outcomes Study*, traduzida e validada para o português. Para avaliação da fragilidade, utilizamos a proposta de Johansen et al. (2007), sendo os sujeitos classificados em frágeis e não frágeis. A comparação entre os grupos foi realizada por ANOVA one-way com análise *post hoc* de Tukey e pelo teste de qui-quadrado, conforme o tipo de variável analisada. Além disso, foi realizada a correlação de Pearson ou Spearman para medir a associação entre variáveis identificadas e a fragilidade. Realizamos, também, uma regressão linear simples, ajustada para sexo e idade pelos escores físico e mental do SF-36.

**Resultados**

A média de idade foi 60,5 ± 11,5 anos. Do total de pacientes, 42,6% apresentavam fragilidade. Os grupos foram diferentes entre si quanto à idade, ao sexo e todos os domínios do SF-36, exceto nos aspectos sociais e nos aspectos emocionais. Quando correlacionamos a fragilidade com as variáveis do estudo, encontramos forte associação com todos os domínios da qualidade de vida, exceto nos aspectos sociais e nos aspectos emocionais. Através da análise de regressão ajustada para idade e sexo, houve diferença entre o grupo frágil no escore físico e no escore mental.

**Conclusão**

Dos oito domínios do SF-36, sete foram diferentes entre os grupos de fragilidade e, quando separados pelo score físico e mental, o grupo frágil mostrou-se pior. Além disso, também houve correlação entre fragilidade e os domínios de qualidade de vida, com exceção aos aspectos sociais. Intervenções sobre os componentes da fragilidade são necessários para melhor conduzir o tratamento dos pacientes e melhorar sua qualidade de vida.

## Introduction

Frailty is emerging as a common syndrome among older adults. However, for patients with chronic kidney disease (CKD), frailty has also been observed in younger age groups [1,2]. This observation reflects a decline in physical functioning and an increase in patient vulnerability [3]. Frailty may even indicate an increased risk of adverse health effects such as morbidity and mortality [4], further reducing patients’ quality of life (QOL).

Furthermore, frailty may lead to a worsening of the patient’s QOL, possibly due to a reduction in functional capacity, which increases physical fatigue. Both of these issues reduce the patient’s mobility, thus lowering social interaction and generating dependency, which can be aggravated in people with clinical complications such as those related to CKD.

Some studies have shown that older adults who do not suffer from CKD but exhibit characteristics of frailty (i.e., exhaustion and reduced strength), show evidence of reduced QOL and increased rates of depression [5-8]. Kanauchi et al. [5] assessed the relationship between frailty, QOL, and wellbeing in older people with cardiometabolic risk factors. Lúpon et al. [6] observed frailty and depressive symptoms in patients with heart failure. Bilotta et al. [9] assessed the relationship between frailty and QOL dimensions in an older Italian population and found reduced QOL in frail participants, as did Lin et al. [10] when studying an older Thai population.

However, to our knowledge, previous studies have not explicitly examined relationships between frailty, QOL, and depression in CKD patients. Because frailty is exacerbated and QOL reduced during the progression of CKD [3,11,12], immediate diagnosis of both is necessary for the implementation of therapeutic interventions designed to minimize further complications.

Thus, the goal of the current study was to evaluate the relationship between frailty and QOL in pre-dialysis CKD patients.

## Materials and methods

### Sample

The Research Ethics Committee of the Federal University of Juiz de Fora approved the current study. All patients provided written informed consent for participation in this study. The sample comprised male and female adults (*N* = 61) receiving pre-dialysis treatment for Stages 3–5 CKD. Patients were excluded if they presented with clinical conditions diagnosed by a physician that would impede physical assessment (e.g., severe neurological pathologies, gout, amputations, Parkinson’s disease, chronic obstructive pulmonary disease, neoplasms, human immunodeficiency virus, and severe physical sequelae caused by stroke or deep vein thrombosis). Patients were also deemed ineligible if they exhibited significant cognitive decline, measured using a translated and adapted version [13] of the Mini-Mental State Examination [14].

### Evaluation of kidney function and comorbidities

The diagnosis of CKD and disease stage was established by estimating patients’ glomerular filtration rate (GFR), based on serum creatinine (Cr) dosage, using a formula developed for the MDRD study proposed by the National Kidney Foundation [15]. Diagnosis was also confirmed through documentation of renal parenchymal injury (i.e., proteinuria and/or abnormal glomerular hematuria) existing for a period exceeding three months.

### Frailty

Frailty was evaluated via indicators of muscle weakness (a Short Form-36 Health Survey (SF-36) physical function score ≥ 75 points), exhaustion (an SF-36 vitality score ≥ 55 points), physical inactivity (whether a patient responded “never” or “almost never” to a question about the frequency of physical activity), and/or unintentional weight loss of over 5 kg in the preceding year [1].

The muscle weakness criterion was worth 2 points, while the others were worth 1 point. A patient with a summed score between 0 and 2 points was categorized as non-frail (NF) while a patient with a score of ≥3 points was categorized as frail (F).

### Quality of life

The SF-36 is a shortened version of a questionnaire used in the Medical Outcomes Study. It has been translated and validated for Portuguese respondents in order to assess QOL in this population [16]. The following QOL domains were evaluated: Physical Functioning, Role Physical, Bodily Pain, General Health, Vitality, Social Functioning, Role Emotional, and Mental Health. While the SF-36 has no cut-off point, scores that vary from 0 to 100 are assigned to each domain. Lower scores indicate poorer QOL [17].

Using the Ware Algorithm [18], we transformed the eight domains into two composite domains: a physical component domain (comprising functional capacity, indices of physical functioning, pain, general health, and vitality) and a mental component domain (comprising indices of social functioning, emotional functioning, mental health, general health, and vitality).

### Statistical analyses

We used SPSS version 17.0 (SPSS Inc., Chicago, Illinois) or STATA version 11.0 (StataCorp LP, College Station, Texas) for all analyses.

Patient characteristics, including demographic, clinical, and laboratory characteristics, were presented as means ± SD or frequencies (i.e., percentage of the total). All variables met the assumptions of normality as indicated by the Kolmogorov-Smirnov test. Comparisons between the two groups were performed using independent-sample *t*-tests.

Chi-square tests were performed in order to identify categorical variables associated with frailty, and Spearman’s correlation analysis was used to measure associations between identified variables and frailty.

To evaluate differences in standardized SF-36 scores between frailty groups, we used a multiple regression model with frailty variables. The NF condition was used as a reference. The model controlled for gender and age. Results were expressed as adjusted means, and differences were assessed between these for patients categorized as NF. The significance threshold was 0.05.

## Results

The mean age of the sample was 60.5 ± 11.5 years, and 42.6% of the participants were diagnosed as frail. Table 1 shows the demographic, clinical, and laboratory data for patients, divided into F and NF groups. The groups differed significantly in terms of age (p = 0.009), gender (p = 0.02), and all QOL domains (Physical Functioning: p = 0.0001; Role Physical: p = 0.03; Bodily Pain: p = 0.03; General Health: p = 0.04; Vitality: p = 0.002; Mental Health: p = 0.05) except Social Functioning and Role Emotional. However, they did not differ in race, body mass index (BMI), smoking status, CKD stage, CKD etiology, comorbidities, Cr level, or GFR.

**Table 1:** Details of demographic and clinical data, as well as biochemical parameters, of the total sample (n = 61) and separated by frailty classification in a group of Brazilian patients with pre-dialysis chronic kidney disease

	**Total (n = 61)**	**Non-frail (n = 35)**	**Frail (n = 26)**	**p**
	**Mean ± SD or n (%)**			
Age (years)	60.5 ± 11.5	57.3 ± 11.4	64.9 ± 10.3	0.009*
Female	25 (41.0%)	10 (28.6%)	15 (57.7%)	0.02*
Black phenotype	28 (45.9%)	13 (37.1%)	11 (42.3%)	0.11
Body mass index (kg/m ^2^)	25.9 ± 5.0	26.2 ± 4.0	28.0 ± 5.5	0.16
Smoking status	9 (14.8%)	5 (14.3%)	4 (15.4%)	0.90
Chronic kidney disease stages				0.15
3	24 (39.3%)	17 (48.6%)	7 (26.9%)	
4	25 (42.6%)	12 (34.3%)	13 (50.0%)	
5	12 (19.7%)	6 (17.1%)	6 (23.1%)	
Etiology of chronic kidney disease				0.56
Arterial hypertension	18 (29.5%)	11 (31.4%)	7 (26.9%)	
Diabetes mellitus	11 (18.0%)	4 (11.4%)	7 (26.9%)	
Glomerulonephritis	9 (14.8%)	7 (20.0%)	2 (7.7%)	
Unknown	8 (13.1%)	4 (11.4%)	4 (15.4%)	
Undetermined or other	15 (24.6%)	9 (25.7%)	6 (23.1%)	
Comorbidities				0.10
Arterial hypertension	23 (56.1%)	14 (40.0%)	9 (34.6%)	
Diabetes mellitus	4 (9.8%)	3 (8.6%)	1 (2.6%)	
Other	25 (41.0%)	11 (31.5%)	14 (53.9%)	
None	9 (22.0%)	7 (20.0%)	2 (7.7%)	
Creatinine (mg/dL)	2.7 ± 1.3	2.7 ± 1.5	2.7 ± 1.0	0.89
Glomerular filtration rate (mL/min/ 1.73 m ^2^)	26.8 ± 12.9	28.6 ± 13.1	24.3 ± 12.5	0.19
SF-36 domains				
Physical functioning	67.2 ± 25.3	84 ± 15.3	46 ± 19	0.0001*
Role physical	65.9 ± 38.7	75 ± 35.3	53.8 ± 40.4	0.03*
Bodily pain	68.8 ± 31.7	76.5 ± 26.7	58.4 ± 35.3	0.03*
General health	56.4 ± 25.2	62 ± 23.8	48.9 ± 25.6	0.04*
Vitality	69.5 ± 23.3	77.4 ± 21.6	58.8 ± 21.5	0.002*
Social functioning	85.4 ± 25.7	84.9 ± 28.4	86 ± 22.1	0.87
Role emotional	74.5 ± 37.2	76.1 ± 36.7	72.3 ± 38.5	0.69
Mental health	76.0 ± 22.3	80.8 ± 21.3	69.5 ± 22.5	0.05*

*p < 0.05.

Table 2 shows the association between frailty and demographic, laboratory, and QOL variables. Frailty did not correlate with Cr or GFR but correlated significantly with all QOL domains (Physical Functioning: r = -0.82, p = 0.0001; Role Physical: r = -0.41, p = 0.001; Bodily Pain: r = -0.28, p = 0.03; General Health: r = -0.31, p = 0.001; Vitality: r = -0.57, p = 0.0001; Mental Health: r = -0.36, p = 0.05) except Social Functioning and Role Emotional.

**Table 2:** Correlation between frailty criteria and age, creatinine, glomerular filtration rate, and quality of life domains in a group of Brazilian patients with pre-dialysis chronic kidney disease

	**R**	**p**
Age	0.25	0.05
Creatinine	0.08	0.55
Glomerular filtration rate	-0.15	0.24
Functional capacity	-0.82	0.0001*
Physical aspects	-0.41	0.001*
Pain	-0.28	0.03*
General state of health	-0.31	0.001*
Vitality	-0.57	0.0001*
Social aspects	-0.12	0.36
Emotional aspects	-0.19	0.14
Mental health	- 0.36	0.005*

*p < 0.05.

QOL represents multiple factors related to patients’ perception of their own lives. In an effort to understand which domains were most strongly associated to frailty, we assessed differences between the F and NF groups based on the composite physical and mental SF-36 domains.

**Figure 1:**
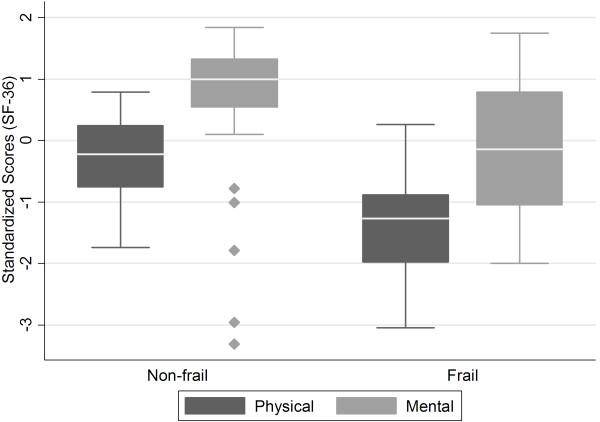
Box plot showing the difference between the non frailty and frailty groups on physical and mental component scores.

As indicated in Table 3 and Figure 1, simple linear regression adjusted for age and gender revealed a significant difference between groups for the physical (cof = -1.12 [-1.47 to -0.76], p = 0.001) and mental (cof = -0.75 [-1.4 to -0.16], p = 0.02) SF-36 components.

**Table 3:** Differences between frailty groups on composite SF-36 physical and mental scores in a group of Brazilian patients with pre-dialysis chronic kidney disease

	**Mental component**			**Physical component**		
	Mean*	**Coeficient**	**p**	Mean*	**Coeficient**	**p**
Non-frail	-1.1	-		-1.12	-	
Frail	-1.81	-0.75 (-1.4; -0.16)	0.02	0.6	-1.12 (-1.47; -0.76)	< 0.001

*Averages adjusted for gender and age in years.

## Discussion

The purpose of the current study was to verify the relationship between QOL and frailty in pre-dialysis CKD patients. The prevalence of frailty in the current sample (42.6%) fell between that documented in other samples (67.7% and 20.9%, [1,3]). It should be noted, however, that the instruments used to assess frailty in previous studies differed from those used in the current study.

As in this study, others have associated frailty with diminished QOL in older adults with various pathologies. These results are similar despite differences in the instruments used to evaluate QOL and frailty.

Kanauchi et al. [5] evaluated 101 older patients with cardiometabolic risk factors and concluded that frail patients had decreased QOL as assessed by the Vulnerability Elderly Survey Index (VESI) and all domains of the World Health Organization QOL (WHOQOL) assessment.

In a cross-sectional study, Bilotta et al. [9] examined older Italian participants from the Study of Osteoporotic Fractures (SOF), which correlated frailty with QOL life domains from the Older People Quality of Life (OPQOL) instrument. Similar to the results in our study, there was no relationship between frailty and QOL domains except social relationships and financial circumstances.

Using the SF-36, Lin et al. [10] analyzed the relationship between frailty through a phenotype proposed by Fried et al. [7] and QOL domains in 933 older participants in Taiwan. The results demonstrated that frailty was associated with all domains except for the physical, emotional, and mental domains. In our study, the only QOL domains that were not significantly associated with frailty were the emotional and social domains; however, there was a relationship between frailty and the physical and mental composite domains.

The relationship between frailty and depression is also relevant. This relationship will be clarified in prospective studies that plan to verify the associations between QOL, depression, frailty, and negative outcomes (e.g., hospitalization and mortality).

Lúpon et al. [6] evaluated the relationship between frailty and depression in 622 hospitalized patients with heart failure who died one year post-assessment. Frailty and depression were associated with worse outcomes. However, only frailty held prognostic value for mortality, even after adjusting for confounds in the multivariate analyses. In their study, the QOL measure—the Minnesota Living with Heart Failure Questionnaire—was not associated with either frailty or depression.

In another analysis, Masel et al. [8] evaluated the relationship between frailty, QOL, and mortality in 1,008 older Mexicans over periods of two and three years. QOL was assessed using the SF-36. Frailty was associated with mortality during the study period; however, this association was attenuated following adjustment of the SF-36 physical domain.

A relationship between QOL and depression in CKD patients has been observed [19-23]. However, previous studies have not assessed the association between frailty and QOL in this patient population.

Regarding the relationship between frailty and QOL, we argue that frailty directly influences QOL by reducing patients’ physical and functional capacity. This, in turn, exacerbates symptoms related to the mental domain.

In CKD, the relationship between frailty and QOL is due to specific factors, including weakness, fatigue, weight loss, low levels of physical activity, social exclusion, mild cognitive changes, and increased vulnerability to stressors [7].

We evaluated depression within a sub-sample of patients in our study (*n* = 37) using the Beck Depression Inventory (BDI). We did not find a relationship between frailty and depression (data not shown).

However, some studies, such as the Netherlands Cooperative Study on the Adequacy of Dialysis (NECOSAD) project conducted by Van Den Beukel et al. [24], have assessed depression by using the mental health and emotional domains or the mental composite score. When the mental health domain was used as an indicator of negative mood, our data confirmed an association between frailty and depression. Chang et al. [25], when evaluating 1,240 patients from the Women’s Health and Aging Studies (WHAS) I and II, recently revealed a similar result.

We also found a difference between frailty groups with respect to age and gender, with the frail group comprising patients who were older and predominantly female. This is similar to the majority of studies that have assessed frailty.

Fried et al. [7] found that age was a contributing factor to the frailty of 5,317 older patients without CKD from the Cardiovascular Health Study (CHS). Women, regardless of age, were more frail then men. Shilipak et al. [26] compared 5,808 older patients with and without CKD and found a higher prevalence of frailty among black women, regardless of CKD status.

In another large study assessing frailty in CKD patients, Johansen et al. [1] observed that frailty was more prevalent in patients on hemodialysis and peritoneal dialysis and was associated with gender (i.e., women were 55% more likely than men to be frail). In addition, this study demonstrated that age was not a reliable predictor of frailty since it was also observed in a significant portion of younger adults.

The current study has some notable limitations. First, we utilized a cross-sectional design, which was restricted to assessing whether there were associations between frailty, QOL, and depression (i.e., no causal inferences can be made). Second, our study employed a small sample recruited from a single treatment center.

We can better manage patient health by exploring the QOL domains that are affected by frailty. Thus, several strategies should be implemented to improve QOL, including physical exercise, treatment of anemia, maintaining hormonal balance, stress management, reduction of inflammation, and treatment for depression.

Additional longitudinal studies evaluating frailty, depression, and QOL among patients with CKD, particularly those undergoing pre-dialysis treatment, are required to clarify these relationships further.

## Conclusion

The data presented in the current study allowed us to conclude that of the eight SF-36 domains, seven were significantly different between frail and non-frail CKD patients. When separated according to physical and mental scores, frail patients performed more poorly. In addition, there were correlations between frailty and QOL domains, excluding indices of social functioning. Therapeutic interventions applied to the components of frailty are needed in order to implement better patient care and improve QOL.

## Competing interests

The authors declare that they have no competing interests.

## Author’s contributions

HNM and FRSG were responsible for the execution of the study and writing the manuscript. FABC and MGB were responsible for statistical analysis and critical review of the study. All authors read and approved the final manuscript.
